# Comparison of three-lobe and en-bloc techniques during the holmium laser enucleation of the prostate learning curve: a prospective, multicenter, cohort study

**DOI:** 10.1590/1806-9282.20252163

**Published:** 2026-06-29

**Authors:** Ozgur Kazan, Yusuf Arikan, Adnan Basaran, Mehmet Zeynel Keskin, Burak Tüfekci, Ilkin Hamid-Zada, Asif Yildirim

**Affiliations:** 1 Istanbul Medeniyet University, School of Medicine, Department of Urology – Istanbul, Turkey.; 2 University of Health Sciences, Tepecik Training and Research Hospital, Department of Urology – Izmir, Turkey.

**Keywords:** Benign prostatic hyperplasia, Laser therapy, Enucleation

## Abstract

**OBJECTIVE:**

The aim of this study was to compare the safety and effectiveness of the three-lobe and en-bloc techniques during the learning curve of holmium laser enucleation of the prostate.

**METHODS:**

We conducted a prospective, parallel-group, multicenter study that included patients who underwent holmium laser enucleation of the prostate between February and December 2024 at two tertiary urology departments. Patients with prostate volumes ≥80 cm^3^ scheduled for holmium laser enucleation of the prostate were alternately assigned (1:1) to either the three-lobe or en-bloc technique. Exclusion criteria included use of anticoagulants, prostate biopsy within the previous month, diagnosis of prostate cancer, or a history of pelvic surgery or radiotherapy associated with overactive bladder symptoms. The study evaluated the performance of a single surgeon at each center using either the three-lobe or en-bloc technique during their holmium laser enucleation of the prostate learning curve. Primary outcomes were enucleation time/efficiency and complications. Secondary outcomes included postoperative incontinence and functional outcomes.

**RESULTS:**

A total of 116 patients were enrolled in the study, with 60 in the three-lobe group and 56 in the en-bloc group. Patients in the en-bloc group were older than those in the three-lobe group (69.4 vs. 64.9 years, p=0.001). Body mass index and performance scores were comparable between groups. The mean prostate volume was 83.5 cm^3^ in the three-lobe group and 81.3 cm^3^ in the en-bloc group (p=0.732). No significant differences were observed in prostate volume or Prostate-Specific Antigen density. Short-term complication rates and postoperative urinary incontinence were similar between techniques. Operative and enucleation times were significantly shorter in the en-bloc group compared to the three-lobe group (86.1 vs. 107.4 min, p=0.042; 56.7 vs. 72.9 min, p=0.001, respectively). However, enucleation and morcellation efficiencies did not differ significantly. Other intraoperative parameters, as well as postoperative functional outcomes and uroflowmetry measurements, were comparable between the groups.

**CONCLUSION:**

The en-bloc technique demonstrated comparable safety and functional outcomes to the three-lobe technique but offered significantly shorter operative and enucleation times during the holmium laser enucleation of the prostate learning curve.

## INTRODUCTION

Surgical approaches for benign prostatic hyperplasia (BPH) have become increasingly diverse, with significant competition among techniques. Among endoscopic methods, transurethral resection of the prostate (TUR-P) has been utilized for many years. More recently, enucleation of the prostate using holmium:yttrium-aluminum garnet (Ho:YAG) laser—known as holmium laser enucleation of the prostate (HoLEP)—has gained widespread adoption globally. As HoLEP becomes more common, numerous technique modifications have been introduced to enhance surgical efficacy, shorten the learning curve, and reduce complications^1,2^. Over the years, various HoLEP techniques have been proposed by different surgeons. Among many, the most commonly practical techniques include the three-lobe, two-lobe, en-bloc, and en-bloc no-touch approaches. The learning curve associated with these techniques switches depending on multiple factors.

The three-lobe technique, originally described and illustrated by Gilling, stands out as a relatively straightforward method due to its anatomical orientation. Many surgeons find it more intuitive, as it involves consistent visualization of the verumontanum, bladder neck, and adjacent lobes throughout the procedure^3^. The en-bloc technique is also in continuous development, and new modifications such as the en-bloc no-touch technique and the early apical release technique are being introduced to increase the effectiveness of the operation and reduce complication rates^4,5^. Recent studies compared the surgical performance (g/kJ/min), efficacy, and safety differences between the two techniques; the en-bloc technique was found to be associated with fewer side effects, shorter enucleation time, shorter laser time, a lower Clavien-Dindo complication grade, and en bloc technique showed four times higher surgical performance efficiency than the other group^6,7^.

The learning curve of HoLEP has been reported to occur at different levels. Still, no definitive number can be remarked for the learning curve of HoLEP. The learning curve may possibly vary depending on multiple factors, including surgeon-related, patient-related, device-related, and surgical technique–related factors. Although some studies have suggested that the learning curve can be completed in fewer than 20 cases, it has most frequently been reported to fall within the range of 20–30 cases, and according to some, within 20–60 cases. Also, the presence of an expert mentor during HoLEP surgery may reduce the learning curve^8,9^. However, the definition of completing the learning curve is not based on uniform criteria across studies. Whether it refers to the surgeon being able to perform the procedure comfortably with confidence, a decrease in complication rates, or the enucleation efficiency reaching a plateau has been addressed using different parameters in the literature^10,11^.

Many factors play a role in achieving the learning curve, and with the integration of newly developed techniques, this period may be prolonged. There is a common belief that achieving orientation is easier in the three-lobe technique. Therefore, in the HoLEP learning curve, it is usually started and continued with the three-lobe technique. However, in enucleation surgeries, the key point is to enter the correct plane between the adenoma and the capsule and to follow that plane. Both methods have specific advantages and disadvantages.

In the present study, we aimed to investigate whether there is a difference between the three-lobe and en-bloc HoLEP techniques during the learning curve by comparing their efficacy and safety in a prospective, multicenter setting. We evaluated the learning curve by assessing both surgeons at the two centers who performed effective enucleation and morcellation, achieving significant postoperative Prostate-Specific Antigen (PSA) reduction, effective voiding, and symptom relief in patients, while also being able to complete the operations confidently. The study was conducted within a structured development program, in which the techniques were applied sequentially in a one-to-one manner.

## METHODS

In this prospective, non-randomized, multicenter comparative cohort study, patients underwent either three-lobe or en-bloc HoLEP in an alternating 1:1 manner at two centers. Although the study was not registered beforehand, perioperative assessments and follow-up were conducted prospectively. After data collection, patient records were combined and analyzed to compare outcomes between the two techniques. Patients who underwent HoLEP between February and December 2024 were included. The study was conducted at two tertiary urology centers located in two different cities in Turkey. Transurethral experience is an important determinant of the HoLEP learning curve; surgeons were selected to have comparable levels of prior transurethral surgical experience. HoLEP procedures were performed by two experienced urologists, each with 8 years of transurethral resection experience; these two surgeons had no prior experience with HoLEP. Both surgeons performed non-blunt dissection and en-bloc no touch techniques. For both surgeons, the first five cases performed using both en-bloc and three-lobe techniques under mentorship were excluded from the study. In one center, a 100 W Cyber Ho Holmium: YAG laser system (Quanta System, Italy) was used, while the other center utilized a 90 W Potent Hz Holmium: YAG laser system (Potent Medical, Guangzhou, China) along with the Hawk® Morcellators (Hawk®, Hangzhou Hawk Optical Electronic Instruments Co., Ltd., Hangzhou, China). In both centers, the procedures were performed using the same configuration settings of 2 J and 45 Hz. Both centers used 26-F continuous-flow resectoscopes.

Patients with a prostate volume of ≥80 cm^3^ who were scheduled to undergo HoLEP were prospectively enrolled in a 1:1 ratio to undergo either the conventional three-lobe technique or the en-bloc enucleation technique. Patients were sequentially allocated regardless of patient characteristics or surgeon preference. Based on previous studies suggesting that surgeons require at least 20 cases to comfortably complete the HoLEP procedure, we aimed to include a minimum of 20 patients per group at each center^12^. Randomization was in an alternating 1:1 sequence by center. Surgeons were blinded to the outcomes of former HoLEPs. Exclusion criteria encompassed current use of anticoagulant medications, a history of prostate biopsy within the preceding month, histologically confirmed prostate cancer, or any previous pelvic surgery or radiotherapy associated with symptoms of overactive bladder. The study specifically assessed the surgical performance of one novice HoLEP surgeon at each participating center, each performing the procedure using their allocated technique as part of their learning curve.

The primary outcome measures were enucleation time, enucleation efficiency (g/min), and the incidence of intraoperative and postoperative complications. Secondary outcomes included the rate of postoperative urinary incontinence and changes in lower urinary tract symptoms and uroflowmetry parameters, assessed before and after surgery. Functional outcomes were assessed preoperatively and at the first month using the International Prostate Symptom Score (IPSS), the Lower Urinary Tract Dysfunction Research Network symptom index (LURN SI-29), the International Index of Erectile Function-5 (IIEF-5), uroflowmetry, and postvoid residual (PVR).

The study was approved by the institutional research ethics committee of the University of Health Sciences, Tepecik Training and Research Hospital, and the study was performed following the ethical standards of the Declaration of Helsinki (ethics approval number: 2025/06-11).

### Statistical analysis

Statistical analyses were conducted using Statistical Package for the Social Sciences version 26 (IBM Corp., Armonk, NY, USA). Normality was assessed by using the Kolmogorov-Smirnov test and the Shapiro-Wilk test. Patient characteristics were evaluated through frequency and percentage for categorical variables. The mean±standard deviation or median (minimum-maximum) was used for descriptive statistics of continuous variables. To compare categorical variables, the chi-square test or Fisher’s exact test was used. For categorical variables with more than 2×2 levels, a Bonferroni adjustment was applied. The Student’s t-test was used to compare continuous variables. A p<0.05 was considered significant.

## RESULTS

A total of 116 patients were included in the study, with 60 in the three-lobe group and 56 in the en-bloc group. Patients in the en-bloc group were significantly older than those in the three-lobe group (69.4 vs. 64.9 years, p=0.001). However, performance status and American Society of Anesthesiologists scores were comparable between the two groups. The mean prostate volume was 83.5 cm^3^ in the three-lobe group and 81.3 cm^3^ in the en-bloc group (p=0.732). There was no significant difference in short-term complications between the groups. The most common short-term complication in the three-lobe group was urinary tract infection (UTI) (3 patients, 5%), whereas prolonged hematuria was the most frequent complication in the en-bloc group (six patients, 10.7%). Postoperative stress and urgency urinary incontinence rates were similar between the two groups (Table 1).

**Table 1:** Patient and postoperative characteristics.

	Three-lobe n=60	En-bloc n=56	p
Age, years, mean±SD	64.9±5.4	69.4±6.6	**0.001**
BMI, kg/m^2^, mean±SD	27.3±4.1	27.2±4.1	0.892
ECOG performance score			
0	37 (61.7)	36 (64.9)	0.564
1	15 (25)	10 (17.5)	
2	8 (13.3)	10 (17.5)	
ASA score			
1	5 (8.3)	4 (7)	0.960
2	40 (66.7)	39 (68.4)	
3	15 (25)	14 (24.6)	
PSA, ng/mL, mean±SD	6.2±5.1	6.5±5.1	0.817
Prostate volume, cm^3^, mean±SD	83.5±31.9	81.3±38.4	0.732
PSA density, ng/mL^2^, mean±SD	0.08±0.05	0.08±0.04	0.518
Short-term postoperative complication			
None	53 (88.3)	46 (80.7)	0.460
Acute urinary retention	2 (3.4)	1 (1.8)	
Urinary tract infection	3 (5)	3 (5.3)	
Gross hematuria	2 (3.3)	6 (10.7)	
Urethral stricture	0 (0)	1 (1.8)	
Postoperative stress UI	8 (13.3)	6 (10.5)	0.640
Postoperative urgency UI	4 (6.7)	4 (7)	0.940

ASA: American Society of Anesthesiologists, BMI: body mass index, ECOG: Eastern Cooperative Oncology Group performance status, SD: standard deviation, UI: urinary incontinence; PSA: Prostate-Specific Antigen. Chi-square and independent samples t-tests were used. Bold values indicate statistically significant results (p<0.05).

Conversion to TUR was required in five patients in the three-lobe group and in one patient in the en-bloc group (p=0.112). The intraoperative complication rates were comparable between the groups. The total operative time (mean 86.1 vs. 107.4 min, p=0.001) and enucleation time (56.7 vs. 72.9 min, p=0.001) were significantly shorter in the en-bloc group. Morcellation time was similar in both groups (18.1 vs. 19.6 min, p=0.256). Likewise, enucleation efficiency (0.91 vs. 1.09 g/min, p=0.074) and morcellation efficiency were comparable between groups (3.50 vs. 3.31 g/min, p=0.636). Catheter removal time and hospitalization duration were also similar (Table 2).

**Table 2:** Operation characteristics.

	Three-lobe n=60	En-bloc n=56	p
Conversion to transurethral resection	5 (8.3)	1 (1.8)	0.112
Intraoperative complication			0.561
None	56 (93.3)	51 (91.1)	
Capsular perforation	3 (5)	4 (7.1)	
Subtrigonization	1 (1.7)	1 (1.8)	
Operation time, min, mean±SD	107.4±36.9	86.1±26.5	**0.001**
Enucleation time, min, mean±SD	72.9±24.8	56.7±19.1	**0.001**
Morcellation time, min, mean±SD	18.1±6.9	19.6±7.4	0.256
Laser time, min, mean±SD	42.0±14.5	43.3±16.9	0.764
Enucleation efficiency, g/min, mean±SD	0.91±0.61	1.09±0.50	0.074
Morcellation efficiency g/min, mean±SD	3.50±2.36	3.31±2.05	0.636
Catheterization time, days, mean±SD	1.97±2.07	1.82±2.02	0.716
Hospitalization, days, mean±SD	1.90±0.69	2.43±2.50	0.119

SD: standard deviation. Chi-square, Bonferroni adjustment, and independent samples t-tests were used. Bold values indicate statistically significant results (p<0.05).

Preoperative and postoperative PSA levels did not differ significantly between the groups. To evaluate functional outcomes, IPSS, LURN SI-29, IIEF-5, and uroflowmetry parameters were assessed. In the first postoperative month, IPSS scores (9.01 vs. 11.75, p=0.188) and quality of life scores (2.55 vs. 2.75, p=0.798) were similar between groups. The mean maximum flow rate (Qmax) was higher in the three-lobe group (9.77 mL/s) than in the en-bloc group (8.25 mL/s); however, 12 of the 116 patients had an indwelling catheter preoperatively. No patients required catheterization postoperatively. At the first postoperative month, Qmax values were similar between groups (16.56 vs. 12.02 mL/s, p=0.063) (Table 3).

**Table 3:** Preoperative and postoperative outcomes.

			p				p
Preoperative IPSS	Three-lobe	22.09±4.46	0.713	Postoperative 1st mo IPSS	Three-lobe	9.01±3.286	0.188
	En-bloc	22.41±4.527			En-bloc	11.75±5.910	
Preoperative LURN SI-29	Three-lobe	20.22±7.206	0.744	Postoperative 1st mo LURN SI-29	Three-lobe	11.55±6.138	0.383
	En-bloc	19.80±6.141			En-bloc	14.50±9.288	
Preoperative IIEF-5	Three-lobe	13.41±4.373	0.951	Postoperative 1st mo IIEF-5 score	Three-lobe	14.09±5.558	0.261
	En-bloc	13.35±5.629			En-bloc	11.92±3.288	
Preoperative IPSS QoL score	Three-lobe	5.10±0.759	0.717	Postoperative 1st mo QoL score	Three-lobe	2.55±1.635	0.798
	En-bloc	5.15±0.751			En-bloc	2.75±2.094	
Preoperative Qmax (mL/sn)	Three-lobe	9.77±3.357	**0.024**	Postoperative 1st mo Qmax (mL/sn)	Three-lobe	16.567±6.269	0.063
	En-bloc	8.25±2.790			En-bloc	12.027±4.596	
Preoperative Qave (mL/sn)	Three-lobe	4.58±1.495	**0.015**	Postoperative 1st mo Qave (mL/sn)	Three-lobe	9.392±2.678	0.091
	En-bloc	3.01±1.411			En-bloc	7.273±3.053	
Preoperative PVR	Three-lobe	142.1±95.49	0.345	Postoperative 1 mo PVR	Three-lobe	40.13±33.31	0.992
	En-bloc	106.7±83.19			En-bloc	40.01±31.94	
Preoperative Hb	Three-lobe	15.58±5.597	0.621	Early postoperative Hgb	Three-lobe	13.13±1.255	0.928
	En-bloc	15.02±5.118			En-bloc	13.16±1.569	
Preoperative PSA	Three-lobe	6.2±5.1	0.817	Postoperative 1 mo PSA	Three-lobe	1.11±0.75	0.967
	En-bloc	6.5±5.1			En-bloc	1.09±1.18	

Hb: hemoglobin; Mo: month; PVR: post-void residual; QoL: quality of life; PSA: Prostate-Specific Antigen; IPSS: International Prostate Symptom Score; LURN SI-29: Lower Urinary Tract Dysfunction Research Network symptom index; IIEF-5: International Index of Erectile Function-5; IPSS: International Prostate Symptom Score. Independent samples t-test was used. Bold values indicate statistically significant results (p<0.05).

## DISCUSSION

**Figure 1:**
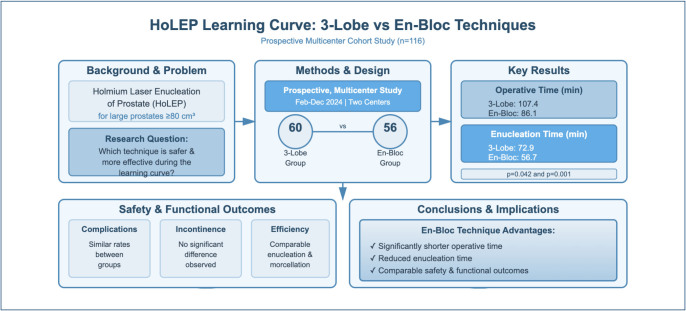
Learning curve of holmium laser enucleation of the prostate: comparison between three-lobe and en bloc techniques.

In our study, we investigated whether there was any difference in efficacy and safety between the two HoLEP techniques—three-lobe and en-bloc—performed sequentially on each case during the learning curve by two surgeons from different centers with similar surgical experience. In the en-bloc HoLEP group, we found a significantly shorter operative time and enucleation time. Enucleation efficiency and morcellation efficiency were similar. Considering that prostates of similar size were included in the study, these results can be interpreted as indicating that at least adenomas of comparable size were enucleated in a shorter period of time (Figure 1).

When comparing the two techniques during the learning curve, perioperative complications, conversion to TUR, short-term complications, postoperative incontinence, operative characteristics, and postoperative functional outcomes should be evaluated. In our study, all these parameters were assessed separately for the two groups. Operative time and enucleation time were lower in the en-bloc HoLEP group. Similarly, studies comparing the three-lobe and en-bloc HoLEP techniques have also reported the en-bloc technique to be faster and more efficient. However, the surgeons in those studies published their results after completing a series of 600–1,396 patients^6,7^. The fact that similar outcomes—namely, faster procedures with comparable efficacy and safety—can be achieved with the en-bloc technique even during the learning curve demonstrates its feasibility. This method could also serve as a starting approach for the HoLEP learning curve in mentorship programs.

In the study by Press et al., three-lobe HoLEP was performed in 49 patients and en-bloc HoLEP in 46 patients by a single surgeon during the learning curve. Prostate volumes of similar size to those in our study were selected. A shorter operative time was found in the en-bloc group, and operative efficiency was also reported in favor of the en-bloc technique. Likewise, complication rates were reported to be very low and comparable between the two groups.

Unlike our study, it is unclear how these two techniques were sequenced or whether any structured program was implemented during the learning curve^13^.

Wang et al. also demonstrated the feasibility of the en-bloc technique in a learning curve of 132 patients. In this study, a single surgeon performed en-bloc HoLEP with the early apical release technique, dividing the cases into two groups: the first 50 patients and the subsequent 82 patients. They reported that the learning curve was completed with this method after 20–30 cases^14^.

In both techniques, we encountered similar rates of intraoperative complications such as capsular perforation or subtrigonization. In almost all other studies, complications have been reported as postoperative events. Since a decrease in intraoperative complications was also considered an indicator of the learning curve in our study, these were evaluated as well. Among postoperative complications, transient incontinence stands out as the most troublesome issue following HoLEP, particularly during the learning curve. In the study by Rücker et al., transient incontinence was observed in 5% of the en-bloc group and 5.5% of the three-lobe group, with the assessment conducted at 3 months^7^. In our study, this evaluation was performed at 1 month, and the rates were 13.3 and 10.5%, respectively, showing no significant difference between the two groups. It should be noted that our assessment was carried out earlier and during the learning curve. In the study by Press et al., the 3-month incontinence rates were higher compared to ours. We think that the difference in incontinence rates observed in our study may be related to patient selection^13^.

Our study is the first to compare, in detail and within a structured program, the sequential application of the three-lobe and en-bloc techniques during the learning curve. We included standardized surgical settings and comprehensive functional and operative outcomes assessed with validated instruments. The inclusion of only patients with a prostate volume ≥80 cm^3^, which ensured a homogeneous patient cohort, allowed a more reliable comparison between the three-lobe and en-bloc HoLEP techniques. One of the main limitations of our study is that, although it was a prospectively followed study, the Consolidated Standards of Reporting Trials criteria regarding randomization and allocation were not followed. The definition of the learning curve cannot be confined to a specific number, as learning is a continuous process. With a higher number of cases, statistically significant results regarding efficiency may also emerge. Inclusion of only two surgeons and use of different laser platforms between centers may limit generalizability. Another limitation is the absence of postoperative 3-, 6-, and 12-month outcomes of the study.

## CONCLUSION

In this prospective comparison of three-lobe and en-bloc HoLEP techniques during the learning curve, both approaches demonstrated comparable safety and functional efficacy. The en-bloc technique provided significantly shorter operative and enucleation times, suggesting that either technique can be safely adopted in structured HoLEP training programs.

## Data Availability

The datasets generated and/or analyzed during the current study are available from the corresponding author upon reasonable request.
